# Invaginating Structures in Mammalian Synapses

**DOI:** 10.3389/fnsyn.2018.00004

**Published:** 2018-04-05

**Authors:** Ronald S. Petralia, Ya-Xian Wang, Mark P. Mattson, Pamela J. Yao

**Affiliations:** ^1^Advanced Imaging Core, NIDCD/NIH, Bethesda, MD, United States; ^2^Laboratory of Neurosciences, National Institute on Aging, Intramural Research Program, Baltimore, MD, United States

**Keywords:** CA3, horizontal cell, retina, neuromuscular, ephaptic, spinule, cannabinoid, indented

## Abstract

Invaginating structures at chemical synapses in the mammalian nervous system exist in presynaptic axon terminals, postsynaptic spines or dendrites, and glial processes. These invaginating structures can be divided into three categories. The first category includes slender protrusions invaginating into axonal terminals, postsynaptic spines, or glial processes. Best known examples of this category are spinules extending from postsynaptic spines into presynaptic terminals in forebrain synapses. Another example of this category are protrusions from inhibitory presynaptic terminals invaginating into postsynaptic neuronal somas. Regardless of the direction and location, the invaginating structures of the first category do not have synaptic active zones within the invagination. The second category includes postsynaptic spines invaginating into presynaptic terminals, whereas the third category includes presynaptic terminals invaginating into postsynaptic spines or dendrites. Unlike the first category, the second and third categories have active zones within the invagination. An example of the second category are mossy terminal synapses of the hippocampal CA3 region, in which enlarged spine-like structures invaginate partly or entirely into mossy terminals. An example of the third category is the neuromuscular junction (NMJ) where substantial invaginations of the presynaptic terminals invaginate into the muscle fibers. In the retina, rod and cone synapses have invaginating processes from horizontal and bipolar cells. Because horizontal cells act both as post and presynaptic structures, their invaginating processes represent both the second and third category. These invaginating structures likely play broad yet specialized roles in modulating neuronal cell signaling.

## Introduction

**Figure 1:**
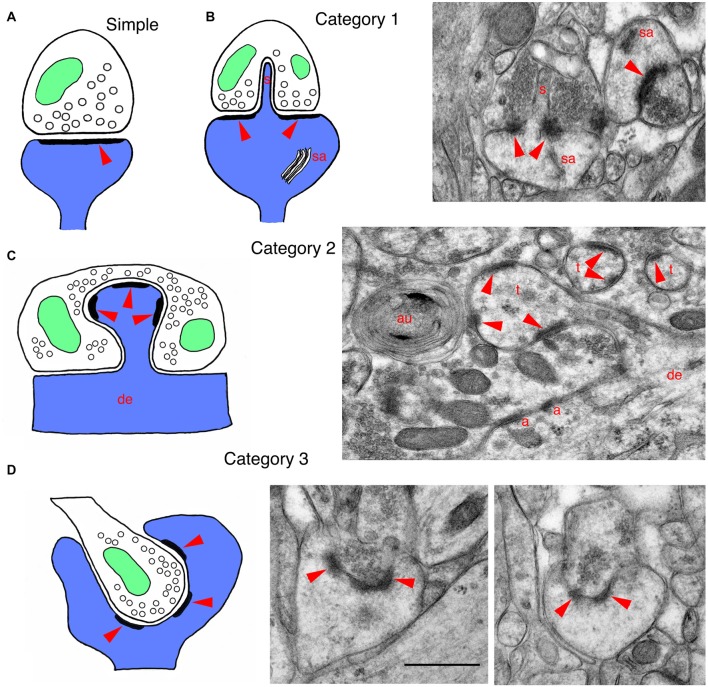
Drawings and EM micrographs illustrating basic examples of the three categories of invaginating structures in mammalian synapses. In all drawings, postsynaptic structures are blue, presynaptic structures are white, mitochondria are green and red arrowheads indicate the postsynaptic densities of synapses. **(A)** A typical or regular postsynaptic spine forming a synapse with a presynaptic terminal. Arrowhead indicates the postsynaptic density (PSD) that is opposite the active zone, where synaptic vesicles fuse with the presynaptic membrane. **(B)** A drawing of a **category 1** invaginating structure shows a large mushroom spine with a spinule (s) that invaginates into the presynaptic terminal; mushroom spines often have a spine apparatus (sa). These large spines with spinules are associated with plasticity and spatial learning. The EM micrograph shows a spinule from a mushroom spine, invaginating into the presynaptic terminal (molecular layer of the dentate gyrus of adult rat). **(C)** A drawing of a **category 2** invaginating structure shows a postsynaptic spine protruding from a dendrite and invaginating into a presynaptic terminal. The EM micrograph was taken from the CA3 region of an adult hippocampus. It shows a Mossy fiber terminal (MFT) forming synapses on the spine-like thorny excrescences (t) extending from the apical dendrites of pyramidal cell neurons. The MFTs also form adherens junctions (a; a.k.a. attachment plaques) with the apical dendrite (de). Note also that a cluster of synaptic vesicles has been enwrapped by phagophores to form an autophagosome (au; Petralia et al., 2011; Vijayan and Verstreken, 2017). **(D)** A drawing of a **category 3** invaginating structure shows a presynaptic terminal invaginating into a postsynaptic process. The EM micrographs were taken from an adult rat dentate gyrus, and show cup spines with partially invaginating presynaptic terminals. The small terminal on the left is almost fully below the edge of the cup, while the terminal on the right is only partially invaginated; in some examples of cup spines described in the literature, the presynaptic terminal can be fully invaginating into the spine (see text for details). Note that tissue for EM in Figures 1, 2 was prepared using freeze substitution, and sections were stained with uranyl acetate and lead citrate (Petralia and Wenthold, 1999; Petralia et al., 2010). Scale bar in the two figures is 500 nm. Animal procedures were performed in accordance with guidelines approved by the institute Animal Care and Use Committee and NIH.

The classic image of a neuronal synapse with a bulbous presynaptic terminal separated from a postsynaptic dendrite shaft or spine (Figure 1A; Shepherd, 2004) is often, in reality, complicated by various invaginating structures. Even sponges, which seem to lack definitive neurons and chemical synapses, can have neuron-like cells with elongate processes making invaginating contacts with other cell processes; perhaps these invaginating contacts represent rudimentary chemical synapses. Some cubozoan jellyfish possess highly developed eyes with photoreceptor synapses that have complex invaginating postsynaptic spines. In fact, almost all major groups of animals, invertebrate and vertebrate, have a variety of invaginating structures at many of their synapses. These invaginating structures can originate from the postsynaptic process, the presynaptic terminal, or glial processes. Many types of invaginating structures do not contain or contact active zones (for example, Figure 1B). These active zone-free invaginating structures have been given various names including spinules, varicosities, and protrusions. In contrast, active zone-associated invaginating structures can be derived from postsynaptic processes that include postsynaptic spines and spine-like structures, or from part or all of the presynaptic terminal. We have previously described three categories of invaginating structures in all animals (Petralia et al., 2015, 2016, 2017). In this short review, we focus on the three categories in mammals, and update the literature. We also discuss how all these invaginations can be essential for precise signaling events among neurons, and contribute to synaptic signaling.

## Examples of Invaginating Structures at Mammalian Synapses

### Category 1. Invaginating Spinules and Related Structures

These invaginating protrusions can be derived from the postsynaptic, presynaptic or glial components of synapses. Although active zones often lie adjacent to the invaginating structures, they do not have any active zone within the invagination (see Figure 1B).

#### Postsynaptic

**Figure 2:**
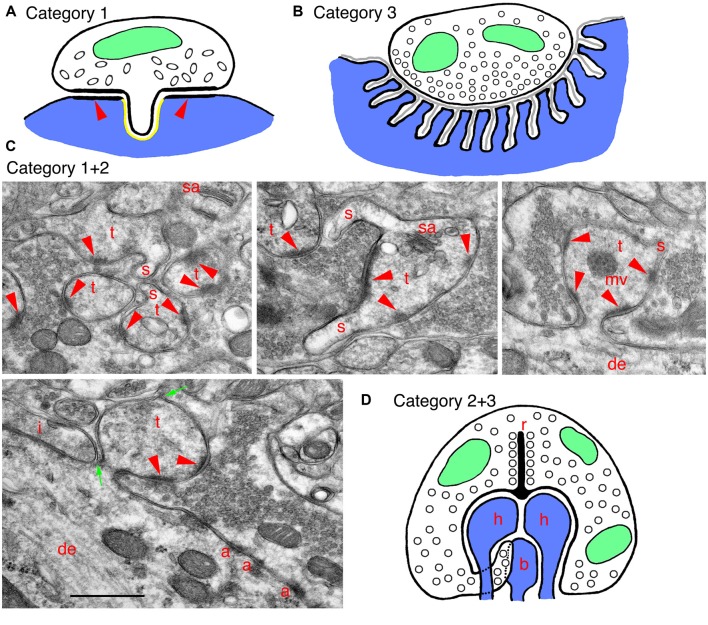
Examples of specialized invaginating structures. **(A)** A drawing of an unusual example of a **category 1** invaginating structure: a presynaptic terminal invaginates into the postsynaptic soma. The synapse has characteristics of inhibitory terminals with a less prominent PSD (arrowheads) and oval-shaped synaptic vesicles. The GABA receptors are on the postsynaptic membrane, lining the PSD (arrowheads) and ringing the invagination, and there are endocannabinoid synthetic enzymes on the postsynaptic side of the invagination (shown in yellow). Endocannabinoid release activates cannabinoid receptors in the presynaptic membrane, and these then mediate retrograde suppression of neurotransmitter release from the terminal. **(B)** A drawing of a specialized example of a **category 3** invaginating structure: a generalized mammalian neuromuscular junction (NMJ). In this example, the presynaptic terminal is only partly invaginated (indented) into the muscle fiber. The indention is lined with deep subjunctional folds in the postsynaptic membrane. A thin basal lamina (gray) extends within the synaptic cleft and into the folds. **(C)** EM micrographs show examples of invaginating structures combining **categories 1 and 2**. These are from the CA3 MFT region, as described in Figure 1C. Thorny excrescences (*category 2*) also commonly invaginate spinules (s; *category 1*) into the MFTs, especially apparent in the upper three micrographs. Note in the left, lower micrograph how thin portions of the MFT shown between the two green arrows surround part of the invaginated thorny excrescence. A tiny spinule is barely visible near the top green arrow. Note also how this MFT isolates the thorny excrescence surface from possible spillover from an adjacent inhibitory terminal (i); the latter is identified by the elongate symmetrical density as well as by some obscure pleomorphic synaptic vesicles (compare to the more distinctive and rounder excitatory synaptic vesicles in the MFTs). Common organelles in the thorny excrescences include the spine apparatus (sa) and multivesicular body (mv). **(D)** A drawing of a specialized invaginating structure combining **categories 2 and 3**. A photoreceptor terminal-synaptic ribbon (r) contacts a deep invagination containing postsynaptic processes (*category 2*) from horizontal (h) and bipolar (b) neurons, as well as projections from the terminal. Rod terminals in mammals usually have a single invaginated ribbon/active zone with two horizontal and two bipolar cell processes, as well as “fingers” of rod cytoplasm, while cone terminals have multiple invaginated ribbon/active zones, each with two horizontal and 1–2 bipolar cell processes (Rao-Mirotznik et al., 1995; Sterling and Demb, 2004; Petralia et al., 2017). The horizontal cell processes also may act as invaginating presynaptic terminals (*category 3*) since they send a retrograde signal to the photoreceptor terminal, mediating a feedback mechanism.

In mammals, postsynaptic spinules have been described best in rat hippocampus, but spinules are found in other parts of the brain such as cerebral cortex and cerebellum (Figures 1, 2; Blanque et al., 2015; Petralia et al., 2015; Familtsev et al., 2016; Rodriguez-Moreno et al., 2017). In the adult rat CA1 stratum radiatum, Westrum and Blackstad (1962) found that spinules are 25–100 nm wide and 75–150 nm long, but Spacek and Harris (2004) found greater variation in size (diameters from <8 nm to 150 nm), with some dendritic spine spinules longer than 0.75 μm; and Tao-Cheng et al. (2009) found postsynaptic spinules as long as 0.5 μm in hippocampal slice cultures. Postsynaptic spinules include those that invaginate into presynaptic terminals, as well as some that invaginate into adjacent axonal or glial processes (Spacek and Harris, 2004). Often the tip of the spinule is surrounded by a coated pit in the opposing cell membrane (hippocampus (Westrum and Blackstad, 1962; Spacek and Harris, 2004; Yao et al., 2005; Tao-Cheng et al., 2009); cerebellum (Eckenhoff and Pysh, 1979)). Spacek and Harris (2004) suggest that: “…spinules provide a general mechanism for signaling and remodeling throughout the brain”. Postsynaptic spinules are involved in synaptic plasticity that occurs during all stages of life, from early postnatal development to old age; one of the best studied examples of synaptic plasticity that involves spinules occurs in large, mushroom-shaped spines of the hippocampus (reviewed in Geinisman et al., 1994; Petralia et al., 2014). Typically, during plasticity such as that initiated by long-term potentiation (LTP), the mushroom spine grows in size (and adds postsynaptic receptor molecules) and a perforation forms in the center of the postsynaptic density (PSD). At the perforation, the postsynaptic membrane may begin to invaginate into the presynaptic terminal as a spinule (Figure 1B); eventually, the PSD may separate into pieces (segmentation) as the spine continues to grow. These spines may go through cycles of enlargement and shrinkage associated with activity and aging. Also, the associated spinules undergo rapid turnover during sustained synaptic activity; this may be a mechanism of membrane retrieval by the presynaptic terminal to compensate for excessive growth of spine membrane induced by activity (Tao-Cheng et al., 2009).

#### Presynaptic

In adult rat hippocampus stratum radiatum, as described above, most spinules originate from postsynaptic structures, but Spacek and Harris (2004) found that about 12% grow from axons and invaginate into other axons or glia. In several regions of the limbic system, such as the globus pallidus, axon terminals can interlock with each other along their lateral surfaces via large processes called pseudopodial indentations (Boyne and Tarrant, 1982). These might function as “variable diffusion traps” that control ions in the extracellular spaces between adjacent terminals, thus probably influencing their membrane potentials. In the dentate gyrus, entorhinal cortex and basolateral amygdala, some inhibitory GABAergic terminals extend short invaginating projections into the postsynaptic neuron; the presynaptic membrane contains cannabinoid receptors, and the invaginating projection opposes a part of the postsynaptic membrane that is rich in an enzyme, DGLα, that synthesizes an endogenous cannabinoid (Figure 2A; Yoshida et al., 2011; Omiya et al., 2015). This structure mediates a retrograde cannabinoid signal producing specific tonic inhibition of synaptic activity. Other interesting examples of presynaptic invaginating structures include thin spinules in early postnatal rodents extending from auditory hair cells and from cerebellar parallel fibers, into postsynaptic afferent processes or Purkinje cell dendrites, respectively (see Petralia et al., 2015). Also, Brusco et al. (2014) shows examples of both presynaptic and postsynaptic spinules in the amygdala.

#### Glial

Glial-derived invaginating projections are common in invertebrates and some lower vertebrates, including at synapses and associated with other parts of neurons (Petralia et al., 2015), but relatively few have been described in mammals. In the cat, Schwann cell processes from the surrounding myelin sheath can extend small invaginating processes into spiral ganglion neurons (Adamo and Daigneault, 1973). Various kinds of glial processes commonly invaginate into axons of mammals (Spencer and Thomas, 1974). One kind involves invaginating “tongues” or “protrusions” originating from surrounding Schwann or oligodendrocyte cytoplasm; these processes appear to ensheath and remove groups of abnormal axonal organelles, and are more common in diseased axons (Spencer and Thomas, 1974). Small spinules also can invaginate into axons from surrounding glia; these typically end in coated pits in axons; Novotny (1984) suggests that glia utilize these structures to transfer substances essential for axonal function.

### Category 2. Invaginating Postsynaptic Spines

These postsynaptic spines protrude directly into the presynaptic terminal and contain active zones within the invagination (Figure 1C).

The best examples in mammals, in the hippocampal CA3 region and retina, are described separately. Other interesting examples of invaginating spines include: spines invaginating into early-postnatal developing auditory hair cells of the mouse and into giant terminals called endbulbs of Held in the anteroventral cochlear nucleus of the early postnatal cat, some invaginating filopodia-like spines in the red nucleus, and those forming some crest synapses (for details, see Petralia et al., 2016). Note that various structures called filopodia are common in the nervous system; they look like spinules, only are larger—usually >100 nm wide and >1 μm long, and usually are not invaginating. Like spines, filopodia contain actin filaments; in contrast, the content of spinules is generally diffuse and poorly defined, and it may be difficult to distinguish wide spinules from thin filopodia. Some filopodia may mediate synaptogenesis of spine synapses and be important components of synaptic plasticity and learning (Fiala et al., 1998; Ozcan, 2017). Crest synapses are particularly unusual, and consist of a flattened, disk-shaped spine with synaptic active zones on the two sides, either invaginating into a single terminal or having two terminals—one per side; they are found scattered throughout the central nervous system (Akert et al., 1967; Petralia et al., 2016). In addition, afferents to taste bud cells often form spine-like indented or invaginated synapses in many mammals (Royer and Kinnamon, 1988, 1991; Witt and Reutter, 1996); the more deeply invaginating ones are described as finger-like projections or processes (Royer and Kinnamon, 1988, 1991).

### Category 3. Invaginating Presynaptic Terminals

These presynaptic terminals protrude directly into the postsynaptic structure (spine or dendrite) and contain active zones within the invagination (Figure 1D).

A modest variety of invaginating presynaptic terminal structures occur, including in developing auditory nerve endbulbs on neuron somas in the cat, vestibular nerve terminals on neuron somas of the rat lateral vestibular nucleus, crested dendrites in the rat interpeduncular nucleus, and cup-shaped spines (see Petralia et al., 2017); also, terminals often partially invaginate (deep indention) into neuron somas in the monkey lateral geniculate nucleus (Saavedra et al., 1968). The crested dendrite is a unique dendritic structure containing several crest spines joined with invaginating presynaptic terminals, found in the interpeduncular nucleus of the rat (Murray et al., 1979). A number of studies have described cup-shaped spines in the cerebral cortex and hippocampus of mammals. Basically, the spine appears to wrap around the smaller presynaptic terminal; the best examples are seen in the rat hippocampal dentate gyrus (Figure 1D; Desmond and Levy, 1983; Frotscher and Léránth, 1986). Presence of cup spines may be affected by neuronal plasticity and they may be more frequent in slice and neuronal cell cultures (Mitchell et al., 2012; Petralia et al., 2017; and unpublished data).

#### Neuromuscular and Secretomotor Endings

In neuromuscular junctions (NMJs) of most animals, invertebrate and vertebrate, presynaptic terminals are indenting or invaginating into muscle fibers; thus, some kinds of terminals are found in a shallow, elongate indention (“gutter”) on the surface of the fiber, while others are invaginating completely into the fiber (Figure 2B; Petralia et al., 2017). Most skeletal muscles in mammals have twitch fibers, defined by their ability to propagate an action potential along the fiber from the NMJ. In skeletal muscles of mammals (and in vertebrates in general), the NMJ postsynaptic membrane (muscle fiber) is often highly folded into subjunctional folds. This is designed to separate the acetylcholine neurotransmitter receptors on the crests of the folds from the sodium channels at the bottom of the folds, as well as align the receptors with the presynaptic active zones (York and Zheng, 2017). The overall arrangement serves to amplify the response to a relatively small amount of neurotransmitter; this is especially efficient in humans compared to mice and rats, since humans have a relatively smaller NMJ area and larger area of folds compared to mice and rats (and even more so compared to frogs; Martin, 1994; Slater, 2008). In addition, an increased depth of the indention or invagination appears to be tied somewhat to greater depth and complexity of the subjunctional folds, and this also could be related to the response speed of the muscle fibers (e.g., for fast vs. slow twitch fibers; Ellisman et al., 1976; Petralia et al., 2017). Various other kinds of muscle fibers have NMJs that can be indented or invaginated, including the slow (tonic) muscle fibers of ear and extraocular muscles, and muscle spindles, cardiac muscle and smooth muscle in internal organs; they also are found at motor nerve endings in exocrine and endocrine gland cells (reviewed in detail in Petralia et al., 2017).

### Category 1+2. Hippocampal Excrescences

Mossy fiber terminal (MFT) synapses in the CA3 area (and also hilus) of the hippocampus form unusual synapses with invaginating postsynaptic, spine-like processes called thorny excrescences (*category 2*; Petralia et al., 2015, 2016; also Reberger et al., 2018). They seem to be a specialization largely unique to mammals, although some similar structures are present in lizards (reviewed in Petralia et al., 2016). The large excrescences can contain some structures that are usually absent in typical spines, such as mitochondria, multivesicular bodies (Figures 1C, 2C), ribosomes and a few microtubules. The excrescences are plastic structures and can form new invaginating extensions with new active zones following LTP (Zhao et al., 2012). MFTs originate from granule cells of the dentate gyrus. These specialized synapses may have evolved in mammals to mediate higher abilities for pattern separation of episodic memory (Treves et al., 2008; Schmidt et al., 2012). The distinct advantage of the invagination is evident in the MFT-thorny excrescence structure. Basically, it forms a very large, continuous synaptic membrane compartment with multiple active zones and excludes any glial processes. This special enclosed synaptic environment facilitates presynaptic diffusion of calcium, spillover of neurotransmitter to reach postsynaptic receptors at multiple active zones, and the spread of zinc co-released from the synaptic vesicles with glutamate (Li et al., 2001; Rollenhagen et al., 2007). So, the invaginated environment keeps some components in and excludes others. The circuitry is complicated and will not be described here, but this unusual synapse is “designed to have a higher net probability of release than most other cortical synapses…” (Henze et al., 2000). Hints of a similar design can be found elsewhere. Thus, dendritic excrescences in the rat somatosensory thalamus (ventrobasal complex) are multiple-branched spines somewhat simpler than the hippocampal thorny excrescences (Spacek and Lieberman, 1974); a similar arrangement may occur in the hamster dorsal lateral geniculate nucleus (So et al., 1985). They invaginate deeply into the large presynaptic terminal, that is stitched to the dendrite shaft via adherens-like junctions, reminiscent of the hippocampal MFTs.

In adult rats, spinules (*category 1*) are common on excrescences (Figure 2C; Petralia et al., 2011). Spatial (water maze) training increases the size of the excrescences and the number of spinules, and some spinules may even form bridges between individual thorns of the excrescences (Stewart et al., 2005); environmental enrichment also increases growth and complexity of the excrescences (Gogolla et al., 2009). Spinules may appear to form a contiguous sequence of structures with autophagosomes in the MFT (Petralia et al., 2011), suggesting that the spinules are involved in turnover of the excrescence membrane during activity, as suggested by Tao-Cheng et al. (2009) for hippocampus spinules in general. Interestingly, MFT spinules and autophagosomes label prominently with antibodies to the sonic hedgehog (Shh) signaling receptors, patched and smoothened, perhaps indicating a role for Shh in trans-synaptic signaling at the MFT synapse (Petralia et al., 2011); Shh also promotes autophagy in synaptic terminals (Petralia et al., 2013). Similarly, autophagy of the Wnt-signal mediator, disheveled, is implicated in regulation of Wnt signaling (Gao et al., 2010). In fact, the increased growth/complexity of thorny excrescences in mice exposed to an enriched environment is correlated with an increase in Wnt in the CA3; and it is likely that enhanced behavioral experience increases local signaling of Wnt at these synapses (Gogolla et al., 2009).

### Category 2+3. Photoreceptor Terminals

Photoreceptor terminals of many animals, both invertebrate and vertebrate, typically have invaginating postsynaptic processes (Petralia et al., 2016). In the retina of mammals, as for most vertebrates, the presynaptic terminal active zones of the rod and cone photoreceptor terminals usually have a deep invagination (Figure 2D) typically with 3–4 postsynaptic processes (spines or spine-like processes; *category 2*) including two from horizontal cell neurons and one or two from bipolar cell neurons (Sterling and Demb, 2004). The neurotransmitter glutamate is released from vesicles associated with ribbon-shaped, dense presynaptic structures (i.e., the synaptic ribbon) and diffuses to reach various populations of postsynaptic receptors placed at different distances from the active zone; in cones at least, this includes some receptors below the invagination (Haverkamp et al., 2000, 2001; Sterling and Demb, 2004). The complex structure of the invagination thus can serve to separate different receptor populations at various distances to control responses according to activity, glutamate release volume and subsequent extent of spillover.

Interestingly, at least the horizontal cell processes also appear to act as invaginating presynaptic terminals (*category 3*); these can be presynaptic to both the photoreceptor terminal and the bipolar cell processes. It is common to find numerous vesicles in the invaginated horizontal cell processes; good examples are found in rats, monkeys and humans; in addition, there is good evidence that the latter processes can be GABAergic (Petralia et al., 2017). However, definitive synapses between presynaptic horizontal cell processes and postsynaptic photoreceptor plus bipolar processes only have been described in the human (Linberg and Fisher, 1988). There is considerable evidence that horizontal processes provide a feedback mechanism to the photoreceptor cell synapse, but the details of the mechanism are debated; generally, this is believed to involve one or more of the following: GABA, protons (pH) and ephaptic transmission (close-range changes in electrical field; Gardner et al., 2015; Kramer and Davenport, 2015; Chapot et al., 2017). Ephaptic transmission might involve connexin hemichannels; these have been found in horizontal cell processes in fish, but it is not clear if they are present in mammalian horizontal cell processes (Klaassen et al., 2011; Gardner et al., 2015; Kramer and Davenport, 2015). Alternatively, both ephaptic and pH-mediated transmission in horizontal cells could be mediated via pannexin-based channels (Kranz et al., 2013; Cenedese et al., 2017; Chapot et al., 2017). In our own studies, we found preliminary evidence of GABA receptor immunogold labeling between horizontal cell processes and adjacent structures, including rod cytoplasmic fingers (Petralia et al., 2017). While GABA transmission, if it occurs, is assumed to involve postsynaptic GABA receptors on the photoreceptor terminal, some evidence indicates that these are autoreceptors on the horizontal cell processes, and they mediate a pH-based feedback (Hirano et al., 2016).

## Conclusion

Invaginating structures are common at synapses and are associated either with developmental plasticity or are integral to the mature synapse structure. In some cases, like the hippocampal MFTs or NMJs, mammals may show particularly well-developed invaginating synaptic structures, reflecting perhaps evolutionary enhancements in the mammalian brain and in brain-muscle coordination. The three categories differ in structure, but in all cases, the invaginated synapse forms a special, enclosed environment that allows wide movement of neurotransmitters and/or other chemicals while excluding other substances, leading to modifications in neurotransmission or selective chemical signaling between the neurons. The invagination also may be specialized for signaling via ephaptic conduction. This has been studied so far in only a few areas such as the retina, but it is likely a widespread mechanism for synaptic modulation, as noted by Gardner et al. (2015).

## Author Contributions

RSP, MPM and PJY wrote the manuscript and RSP, Y-XW and PJY contributed to the figures.

## Conflict of Interest Statement

The authors declare that the research was conducted in the absence of any commercial or financial relationships that could be construed as a potential conflict of interest.
